# Large-scale patterning of single cells and cell clusters in hydrogels

**DOI:** 10.1038/s41598-018-21989-4

**Published:** 2018-03-01

**Authors:** Xiangyu Gong, Kristen L. Mills

**Affiliations:** 1 Department of Mechanical, Aerospace, and Nuclear Engineering, Rensselaer Polytechnic Institute, 110 8th St, Troy, NY 12180 USA; 2 Center for Biotechnology and Interdisciplinary Studies, Rensselaer Polytechnic Institute, 110 8th St, Troy, NY 12180 USA

## Abstract

Biophysical properties of the extracellular matrix (ECM) are known to play a significant role in cell behavior. To gain a better understanding of the effects of the biophysical microenvironment on cell behavior, the practical challenge is longitudinally monitoring behavioral variations within a population to make statistically powerful assessments. Population-level measurements mask heterogeneity in cell responses, and large-scale individual cell measurements are often performed in a one-time, snapshot manner after removing cells from their matrix. Here we present an easy and low-cost method for large-scale, longitudinal studies of heterogeneous cell behavior in 3D hydrogel matrices. Using a platform we term “the drop-patterning chip”, thousands of cells were simultaneously transferred from microwell arrays and fully embedded, only using the force of gravity, in precise patterns in 3D collagen I or Matrigel. This method allows for throughputs approaching 2D patterning methods that lack phenotypic information on cell-matrix interactions, and does not rely on special equipment and cell treatments that may result in a proximal stiff surface. With a large and yet well-organized group of cells captured in 3D matrices, we demonstrated the capability of locating selected individual cells and monitoring cell division, migration, and proliferation for multiple days.

## Introduction

Cell behavior is markedly variable not only between populations of cells of different types or from different tissues, but also within a population of cells^1–4^. To understand the extent of variability between or within populations of cells, it is desirous to characterize a large sample of them. Typically, physical measurements on a large number of cells means removing them from physiologically relevant matrices and only capturing data at one time point (i.e., snapshot measurements)^5^. However, it is becoming increasingly apparent that important aspects of cell behavior are elicited by their interactions with the extracellular matrix (ECM)^6–9^. An example of this is the drastic difference in exhibited morphology dependent upon whether cells are plated on a 2D substrate or within a 3D matrix (Figure S1). Therefore, it would benefit a wide variety of studies to have a simple method to pattern cells within 3D matrices for observation of their behavior over extended periods of time (longitudinal).

Embedding cells in a 3D matrix is most simply achieved by mixing cells with a liquid precursor to a synthetic or biological hydrogel and allowing the gelation process to encapsulate the cells. Long-term monitoring of selected single cells or cell clusters in a mid- to high-throughput fashion then becomes a significant challenge, if not impossible, as the cells are positioned randomly. Researchers have resorted to embedding small numbers of cells into a matrix for long-term studies of single-cell behavior, which eases the experimentalist’s efforts to locate cells^7^, but often does not provide a large enough sample set for significant statistical analyses. One way of achieving better statistics on observable cell behavior in 3D culture has been to employ a modified hanging drop protocol. Using a hydrogel precursor mixed with cells to form the hanging drops is a simple way to encapsulate cells in controllable positions for high-throughput analyses^10,11^. However, this method only creates macro-scale arrays and is not suitable for single-cell analysis because the number of cells in each drop will vary.

Patterning methods and scaffolds have been devised in order to controllably position single cells or cell clusters for gathering large, longitudinal sets of data. These methods often take advantage of material surface properties, morphologies, or micropatterns to capture cells in fixed positions to promote cell attachment and elicit a mechanobiological response^12–15^. Microwells, for example, can be used to rather simply achieve cell placement^16–19^. Furthermore, they have not only been used as a niche where cells may proliferate, but they have also been used as a tool for transferring cells into other 2D environments^20,21^. Surface acoustic waves have been used to move single cells to desired positions on a 2D substrate^22^. Engineered scaffolds, such as polymer structures fabricated via direct laser writing (DLW)^23^ and crack-based patterning^24^, provide single cells with adhesive, topological supports in a 3D space. Whereas these methods allow for cell anchorage and ease of locating and image collecting, the stiff and/or 2D nature of the substrates (e.g., glass or plastic surfaces, 2–4 GPa) do not provide an accurate analog to the soft, 3D nature of the *in vivo* environment (e.g., breast tissue, hundreds of Pascals; human intestinal tissue, thousands of Pascals)^25,26^.

In between 2D and 3D patterning methods are overlay methods, where cells are patterned on a substrate and then covered with a layer of hydrogel or other 3D matrix. Some innovative methods to manipulate cells into patterns include anchoring DNA-labeled cells on a DNA-patterned substrate^27^ and using dielectrophoretic (DEP) forces to attract cells to patterned nodes^28–30^. After the cells are positioned, a layer of hydrogel may be formed on top. Researchers have also used an array of magnetic nodes to trap magnetically labeled cells in between two layers of collagen^31^. Position control over cell placement is indeed accurate, however these methods require special tools (e.g., molecular printing, gold coated nodes, specially treated cells) not easily accessible in every lab. Another drawback of some of these methods for mechanobiological experiments is the presence of stiff substrates and/or interfaces necessitated by the patterning methods, which may prevent the full encapsulation of the cells.

Fully embedding a large population of single cells at specified locations in a 3D, uniform, and soft environment presents a particular challenge. No method has yet been devised in which single cells or small cell clusters may be accurately patterned, without using any externally applied forces or chemical treatments, in various soft and continuous 3D matrices, allowing for dynamic studies of individual cells’ responses to biomechanical stimuli. The goal of this work was to develop a simple and low-cost platform for patterning a large population of single cells or cell clusters completely within a 3D environment (i.e., without any undesirable discontinuous or stiff boundaries). The resulting platform should allow for longitudinal observation of the single cells or cell clusters and control over the cell-cell distance within various cell array patterns. In this article, we describe the design, fabrication, method, and performance of the platform that we developed.

## Results

Our platform capitalizes on the single-cell arraying power of microwells, which are fabricated by standard soft lithography techniques. Once the cells are trapped in the microwells, the platform is inverted and the cells are allowed to drop into a hydrogel that is in the process of gelation. Therefore, we term the platform “the drop-patterning chip”. In this article, the detailed operation of the drop-patterning chip is first presented for large-scale arrays based on collagen I. Performance of the method is evaluated by its patterning efficiency, single-cell occupancy rate, and spatial distribution of cells in 3D collagen gel. The gentle transfer method furthermore leads to excellent cell viability. Finally, we present longitudinal observations of cell motility and *in situ* development of multicellular tumor spheroids (MCTS) from these individual cells in two representative, widely used matrices: collagen I and Matrigel.

### The drop-patterning chip

**Figure 1:**
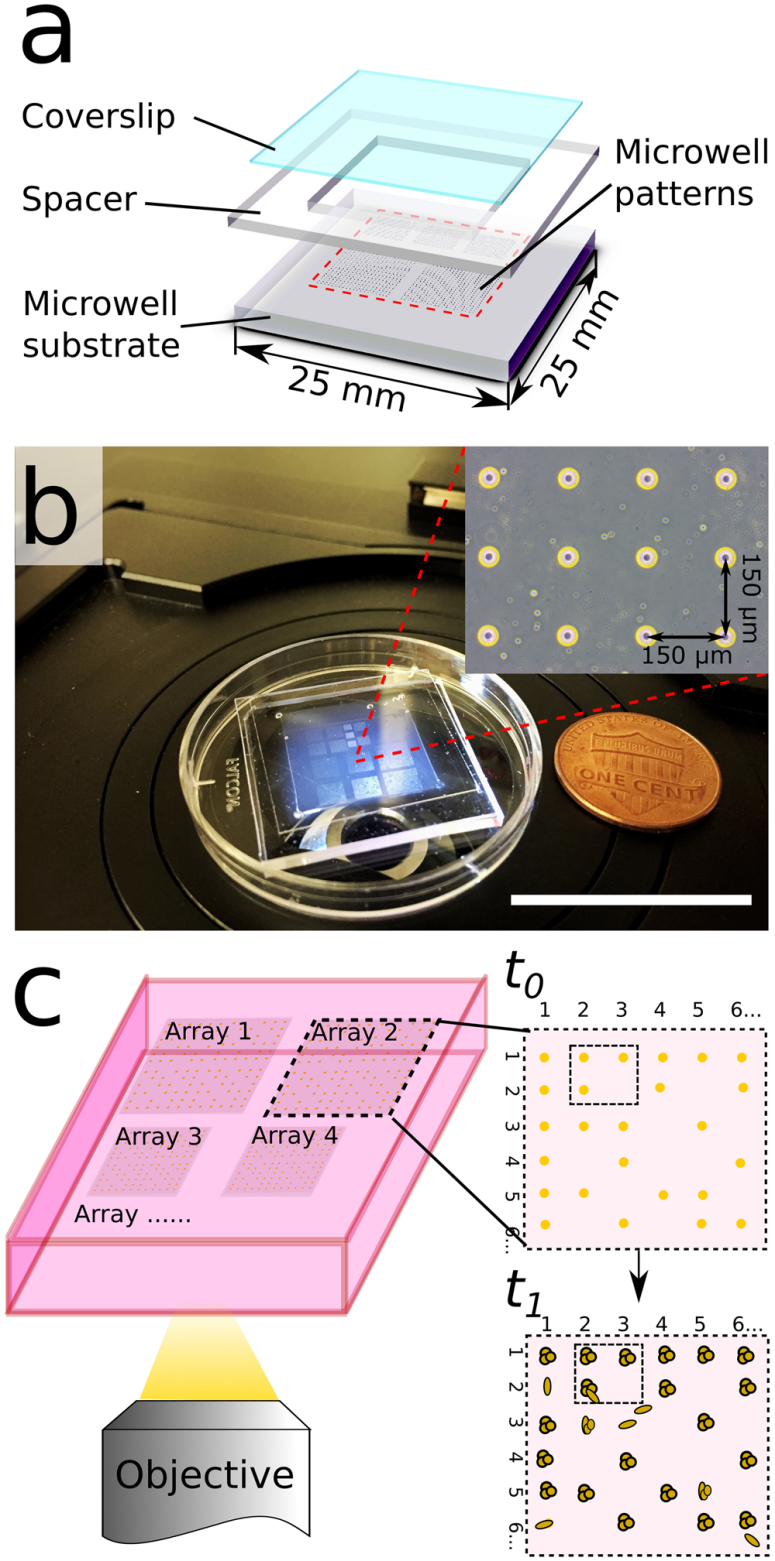
Drop-patterning chip design. (**a**) Schematic of the three-layer configuration of the drop-patterning chip. From bottom to top: PDMS microwell substrate (1.5 mm thick), PDMS square frame spacer (inner side lengths 15 mm, thickness: approximately 600 µm), standard glass coverslip (22-mm square) (**b**) Photograph of an assembled chip with collagen I. The chip was designed to fit in a 35-mm Petri dish lid. The inset shows a section of a microwell array with inter-well spacing of 150 µm. (Scale bar: 25 mm) (**c**) The hierarchical organization of cells patterned in a hydrogel enables repeated tracking and longitudinal observation (time: *t*_0_ to *t*_1_) of a large sample of cells.

The drop-patterning chip consists of three layers (Fig. 1a), which create a closed chamber that is filled with hydrogel (Fig. 1b). The three layers, from bottom to top, are a poly dimethylsiloxane (PDMS) microwell substrate, a PDMS square frame spacer (thickness: approximately 600 µm), and a standard glass coverslip (thickness: 170 µm). Due to the natural adhesion of PDMS to itself and between PDMS and glass, the three layers spontaneously bond together with slight pressure applied by hand. This bond is reversible so the three components can be cleaned and reused. The microwell dimensions—30 µm in diameter and 27 µm in depth—are on the order of the size of a single cell. All data presented here are based on the experiments carried out on microwell arrays with center-to-center spacing (along both columns and rows) of 100 µm, 150 µm, or 200 µm. On each drop-patterning chip, we designed four arrays with 100-µm spacing, fours arrays of 150-µm spacing, and three arrays of 200-µm spacing. Each array is comprised of 400 microwells (20 × 20). Hundreds to thousands of cells may be captured in the wells on a chip for subsequent simultaneous patterning in a hydrogel (Fig. 1c).

### Step-by-step drop-patterning in collagen

**Figure 2:**
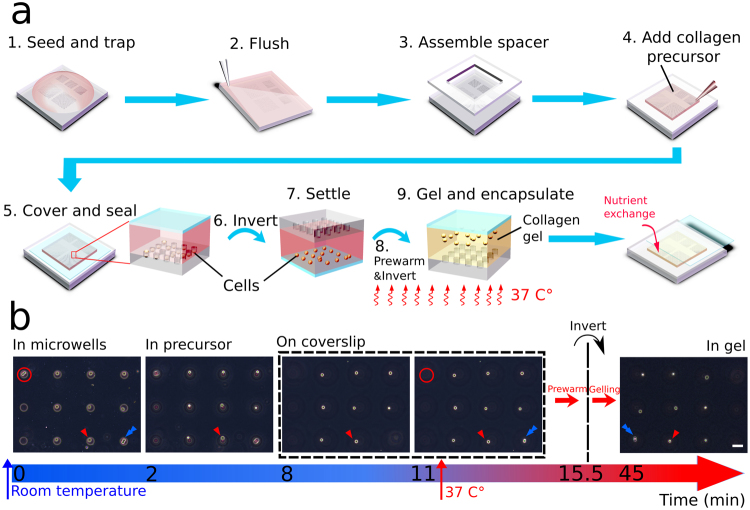
Schematics and images to illustrate the drop-patterning method. (**a**) 1. A cell suspension is seeded on a substrate containing arrays of microwells. The cells are allowed to settle for 5 minutes. 2. Excess cells are gently flushed away with PBS. 3. A PDMS spacer is assembled onto the microwell substrate to create a chamber. 4–5. Once filled with collagen solution, the entire chamber is sealed with a coverslip. 6. The chip is inverted and the trapped cells begin falling out. 7–9. When most cells settle on the glass coverslip, the chip is prewarmed at 37 °C for 4.5 minutes and then inverted again. The cells start falling back into the collagen precursor and the collagen is allowed to gel at 37 °C. After the cell pattern is encapsulated in the collagen gel, the coverslip is carefully slid aside for nutrient exchange (see also Figure S2). (**b**) On chip, real-time monitoring (bright-field imaging) of cell-array embedding in collagen by drop-patterning. (Movie S1) The imaging started immediately after the first inversion, and always focused on a single cell indicated with a red triangle. The cell doublet in the red circle was stuck in the well and did not fall into the collagen precursor. The blue arrow may be used to keep track of a cell/position before and after the second inversion. Bright-field images of the cells in collagen before and after gelation are presented in Figure S4 to show the structural difference between collagen precursor and collagen gel. (Scale bar: 50 µm).

The first set of steps of the drop-patterning technique includes priming the microwells with cells and assembling the chip (Fig. 2a, steps 1–5). A cell suspension (approximately 250 μL, 1 × 10^6^ cells/mL) was deposited on to the PDMS substrate patterned with microwell arrays. Treating the PDMS substrate with air plasma and then incubating in bovine serum albumin (BSA) prior to seeding promoted surface wetting and prevented cell attachment, respectively. After allowing the cells to settle into the microwells for 5 minutes, excess medium and cells were removed from the surface by gently flushing with phosphate-buffered saline (PBS). The PDMS spacer (middle component, Fig. 1a) was then assembled atop the substrate, creating a chamber. This chamber was filled with about 300 µL collagen I solution (1.0 mg/mL) and then sealed with the coverslip (top component, Fig. 1a).

The second set of steps of the drop-patterning technique transfers the array of cells into the collagen gel (Fig. 2a, steps 6–9). The enclosed chip was inverted and the trapped cells were allowed to fall, due to gravity, out of the microwells. Adhesion of the cells to the PDMS microwell walls varied, therefore not all cells fell out of the microwells simultaneously. This means pattern fidelity may be lost and some cells may not be fully transferred into the gel. To improve the pattern fidelity, we first let the cells fall to the coverslip at room temperature, which took about 10 minutes. The chip, with most cells on the coverslip, was then prewarmed at 37 °C in an incubator for 4 minutes and 30 seconds, allowing the collagen to start gelling. Then we inverted the chip once again upon which time all cells fell from the coverslip simultaneously; adhesive interactions between the cells and the BSA-treated coverslip were weaker and more uniform than those between the cells and microwells. The synergy of the rate of collagen gelation and velocity of the falling cells resulted in the cell array becoming fully encapsulated approximately on the same focal plane. After the cells were patterned in collagen gel, the whole chip was submerged in medium for on-chip 3D cell culture. PDMS chambers have been proved to be suitable for 3D cell culture due to the oxygen permeability of PDMS^32^. The coverslip was also carefully slid aside for nutrient and oxygen exchange (Figure S2).

To visualize the drop-patterning method, the whole process was monitored under a bright field microscope (Zeiss, Axio Vert.A1) (Fig. 2b and Movie S1). Focus was maintained on a single cell (indicated with red triangles) in the array. Immediately after the first chip inversion (labeled Time 0), the cells were still trapped in the microwells. After 2 minutes, the microwells were out of focus, which means cells left the microwells and were traveling through the collagen solution. It took approximately 8 minutes for the single cells and 11 minutes for most the cells and cell clusters (i.e., a doublet of cells, indicated with a blue arrow) to settle on the coverslip. Another doublet remained stuck in a microwell (indicated with a red circle). Then the chip was transferred into an incubator for prewarming. After an incubation time of 4 minutes and 30 seconds at 37 °C, the chip was inverted a second time and incubated at 37 °C for 30 minutes longer. The resulting, fully embedded, 3 × 3 cell array is shown in the final image of Fig. 2b. We verified the cell arrays were fully embedded in the gel-filled chamber without contacting either top or bottom surface by imaging the chip on different focal planes after the collagen gelled (Figure S3).

### Cell patterning efficiency

Cell trapping efficiency is considered an important measure of the performance of microwell arrays in high-throughput research on anchorage dependent cells^17,33^. Similarly, the percentage of cell-occupied positions in the embedded 3D pattern reflects the overall efficiency of the drop-patterning method, which we defined as cell-patterning efficiency. As shown previously, not all trapped cells fell out of the wells, which means there was a nominal discrepancy between the cell-trapping efficiency in the microwells and the cell-patterning efficiency in collagen. Both the cell-trapping efficiency and cell-patterning efficiency were calculated according to equation (1), by dividing the total number of cell-occupied positions either in the microwells or in the collagen by the total number of microwells.

1 $${\rm{Efficiency}}\,( \% )=\frac{\text{Number}\,\text{of}\,\text{Occupied}\,\text{Positions}}{\text{Total}\,\text{Number}\,\text{of}\,\text{Microwells}}\times \,100$$

**Figure 3:**
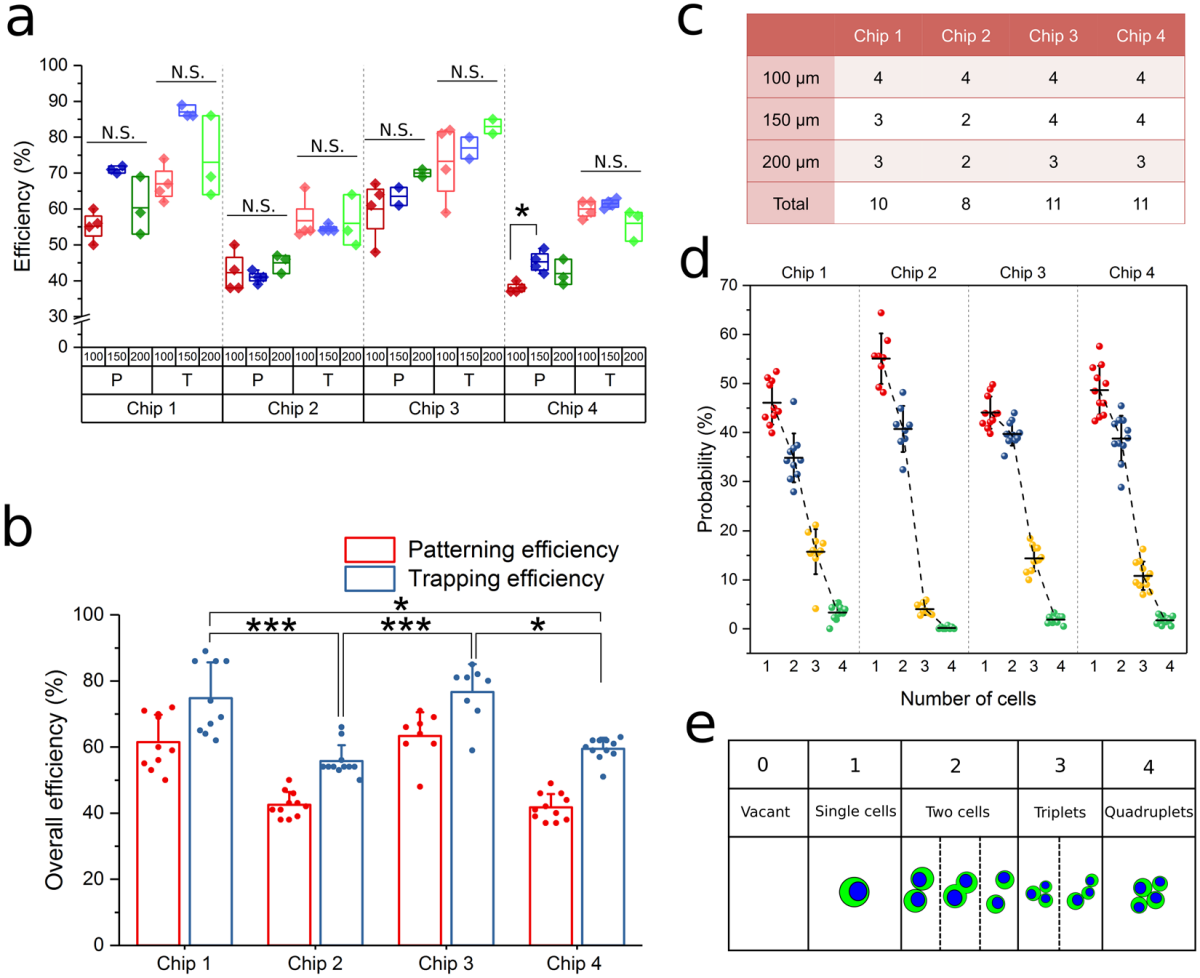
Cell trapping and patterning efficiencies of the drop-patterning method. (**a**) Trapping efficiency (T) and patterning efficiency (P) of arrays with inter-well spacing of 100 μm, 150 μm, and 200 μm on four chips. Kruskal-Wallis test with Dunn’s post hoc for multiple comparisons was performed between efficiency data sets within individual chips. Boxes represent 25th to 75th percentile and whiskers represent minimum-maximum. No statistical difference (N.S.) occurred in the efficiencies between the different inter-well spacings except in one case. (*P < 0.05). (**b**) Mean efficiencies of all arrays on each chip were depicted on a bar plot (mean ± s.d.) overlaid with data (*P < 0.05, ***P < 0.001 based on Kruskal-Wallis test with Dunn’s post hoc for multiple comparisons). (**c**) The number of arrays used to measure efficiencies on each chip. (**d**) The probability distribution of number of cells per occupied position determined from observations of all 20 × 20 arrays on the four chips. (**e**) Schematics illustrating the observed cell occupancy scenarios at the drop-patterned positions.

Cell-trapping (T) and cell-patterning (P) efficiencies for this method were measured on a total of forty 20 × 20 microwell arrays distributed over four chips (Fig. 3a). All three different inter-well spacings (100 µm, 150 µm, and 200 µm) were used. Figure 3c summarizes the number of arrays that were measured on each chip. No statistical difference was measured in the patterning and trapping efficiencies between different inter-well spacings within a chip (Fig. 3a), which suggests that inter-well distance does not markedly affect efficiencies. The discrepancies between patterning and trapping efficiencies on different chips showed consistency regardless of the fluctuation in trapping efficiency (details in Figure S5), which implies that this discrepancy is mainly due to the inherent adhesive interactions between cells and the microwell substrate material. In Fig. 3b, the overall trapping and patterning efficiencies on the four individual chips were calculated as the mean of the efficiencies of all arrays on each chip from Fig. 3a. The difference in trapping efficiency was statistically significant between chips. We attribute this fluctuation in trapping efficiency to the imprecise nature of the manual trapping operations. In other words, increasing the trapping efficiency would lead to a higher patterning efficiency. The average cell-trapping efficiency and cell-patterning efficiency of all arrays on the four chips were 65.7 ± 11.5% and 51.2 ± 11.7%, respectively.

In addition to single cells, small cell clusters, such as doublets and triplets, were trapped in the microwells and patterned in collagen (Fig. 3e). This occurred due to a combination of factors including cell size variation and the susceptibility of cells to dissociation with enzymatic digestion. For the cell line and treatment we used (see Materials and Methods), the probability of finding a given number of cells in an occupied position corresponding to the 20 × 20 positions of an array is presented in Fig. 3d. Each data point present a probability measurement based on an array. The single-cell probability was 40–60% on average and depended on how well the cells were disassociated. Even though cell-patterning efficiency may vary, the probability of the number of occupant cells per position was relatively consistent across chips.

### Large scale array patterning and characterization of spatial distribution in collagen

**Figure 4:**
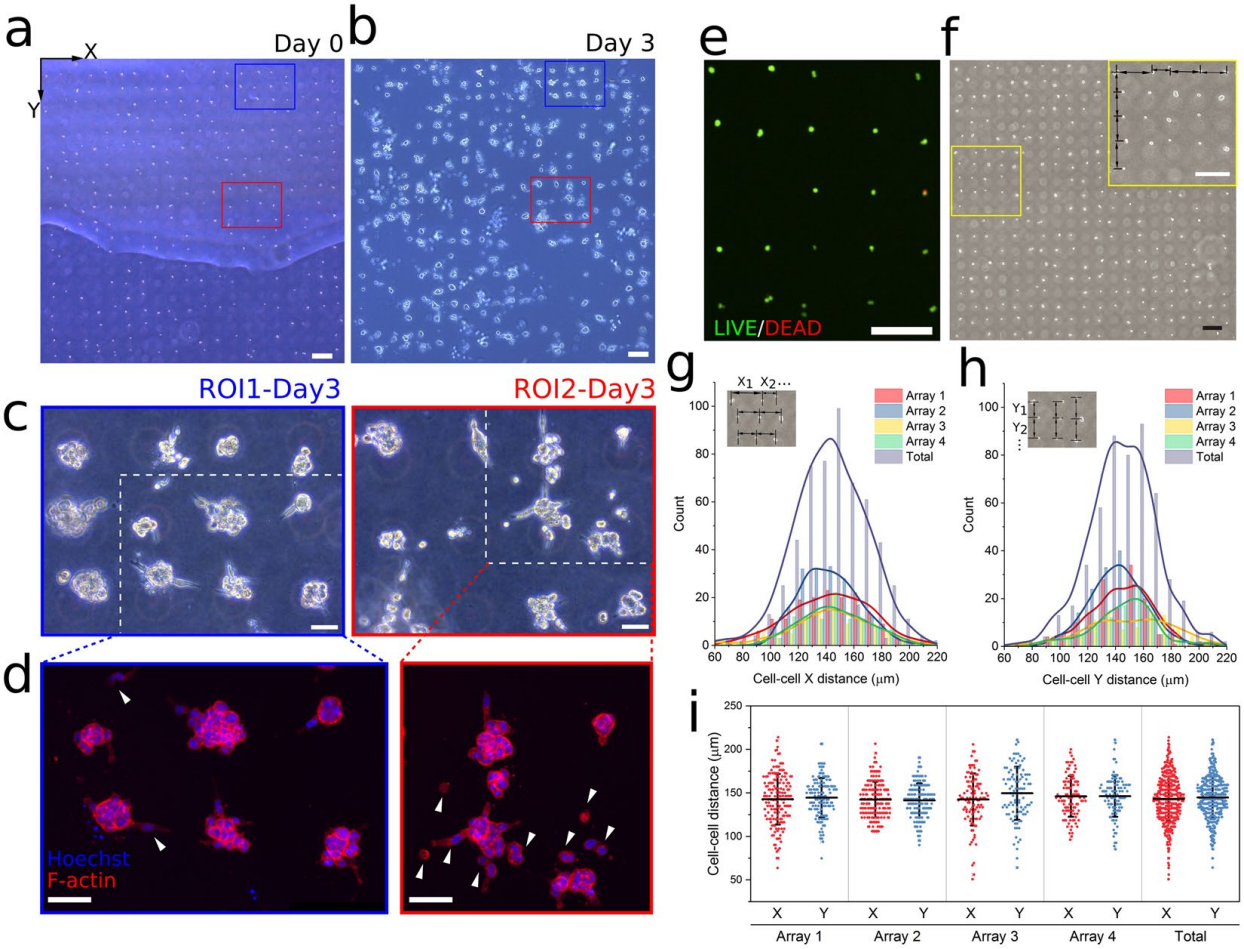
Large-scale cell array patterning in 3D collagen. (**a**) An array (patterned by 20×20 microwell array, inter-well spacing: 150 μm, patterning efficiency: 61%) of single cells and small cell clusters developed into (**b**) an array of MCTS after three days. The air bubble trapped between the PDMS and Petri dish lid in (**a**) did not interfere with drop-patterning. Region of interest 1 (ROI1) and region of interest 2 (ROI2) are enclosed by blue and red lines, respectively. (Scale bars: 200 μm) (**c**) Phase-contrast images of the MCTS array developed from the cells in ROI1 and ROI2 after three days. (Scale bars: 50 μm) (**d**) Immunofluorescence imaging (blue: Hoechst, red: F-actin, maximum projection) of the MCTS array developed from the cells in ROI1 and ROI2 (enclosed in white dotted lines in c) after three days, showing the difference in morphology between MCTS. Cell migration into the collagen matrix is observed (arrow heads). (Scale bars: 50 μm). (**e**) Viability assay (19 hours after embedding) of drop-patterned cells with calcein AM (live cells, green) and EthD-1 (dead cells, red). (**f**) Cell array patterned (patterning efficiency: 65.8%) in collagen by 20×20 microwell array with inter-well spacing of 150 μm in the columns and rows showing how the cell-cell distance characterization was performed (Scale bar: 200 μm). Kernel distributions of cell-cell distances (**g**) in the rows (X) and (**h**) in the columns (Y) on four arrays are overlaid on the corresponding histograms. (**i**) Distribution of all distance measurements overlaid with mean ± standard deviation.

Compared to the common *in vitro* 3D cell culture protocol of randomly embedding hundreds or thousands of cells in a matrix, the drop-patterning chip is able to embed a similar sample size of cells in a matrix, approximately on the same focal plane in a well-organized way (Fig. 4a). The cells may be cultured for time periods up to several days (Fig. 4b). Cells can always be located in an array of hundreds of cells during multi-day incubation times. To illustrate this, we used two ROIs, which are drawn on images at the time of drop-patterning (Fig. 4a) and after three days (Fig. 4b and Figure S6). On day 3, the cells (phase-contrast images shown in Fig. 4c) were fixed and their nuclei and actin fluorescently labeled in order to visualize cell morphology as the result of the 3-day incubation within the drop-patterning chip (Fig. 4d). The majority of cells formed small MCTS with diameters of 30–60 μm. However, there were a few positions where cells exhibited a much more migratory phenotype (e.g., white arrowheads in Fig. 4d). The drop-patterning chip also demonstrated the ease of protein expression assay for the cells in selected positions via immunostaining. For example, the intermediate filament protein vimentin, which is typically upregulated in cells undergoing epithelial-to-mesenchymal transition (EMT)^34,35^, was labeled on the array of MCTS, as shown in Figure S6.

In order to confirm that drop patterning does not damage living cells, cell viability assays were conducted via calcein acetoxymethyl ester (AM) and ethidium homodimer-1 (EthD-1) staining 19 hours after the cells had been patterned in the collagen gel. Figure 4e is a representative image of the resulting fluorescent signals on a section of an array. Two replicates (two chips prepared at different times) following the same drop pattering protocol were tested. Random regions on the chips were imaged. We achieved an average viability of 98%: 458 of 470 cells in the first chip and 358 of 361 cells in the second chip survived.

While cells are being drop-patterned in the collagen solution, cell drifting in the plane of the array may occur. Drifting influences the patterning fidelity (Fig. 4f). To quantify the fidelity of the drop-patterning method, we measured the horizontal (X) and vertical (Y) distances (arrows in Fig. 4f) between two cells or clusters that were patterned by neighboring microwells (150 µm spacing) immediately after collagen gelation. Kernel distributions of the cell-cell distance (Fig. 4g,h) were based on a total of 558 measurements in the X direction and 515 measurements in the Y direction on four arrays from four individual chips. On each array, at least 100 cell-cell spacing distances in each direction were measured. The number of measurements depended on the cell patterning efficiency. The cell-cell distances in both directions in each array are also presented as the mean ± standard deviation overlaid with all measurements in Fig. 4i. Arrays from difference chips showed similar distributions in both directions. The averages and standard deviations of all measurements are 143 ± 26 µm in the X- and 144 ± 24 µm in the Y-direction.

### Longitudinal observations of single cell motility, division, and tumor development in collagen

**Figure 5:**
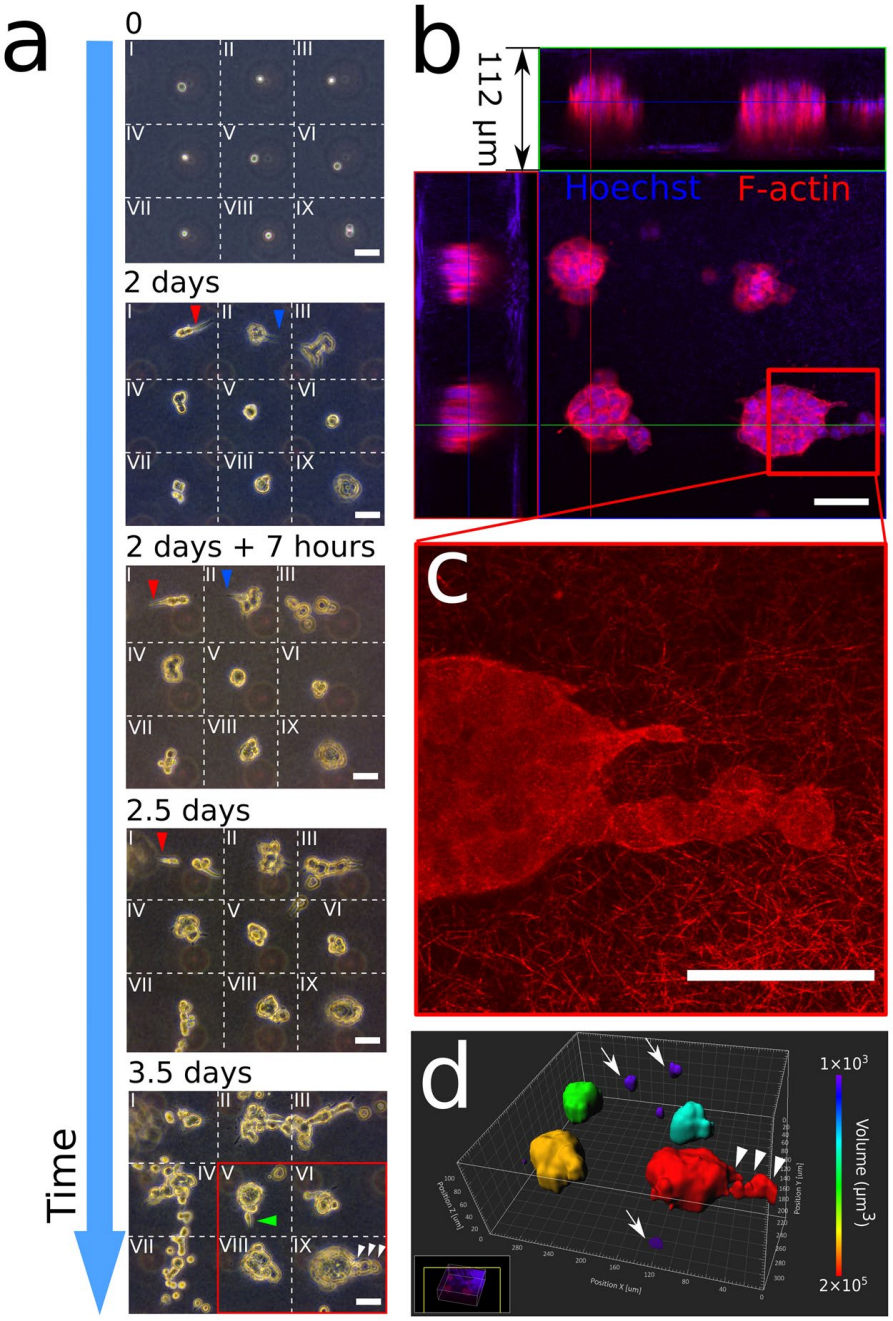
Longitudinal observations of 3D on-chip single cell motility and MCTS development in collagen. (**a**) Phase-contrast images of MCTS progression from a 3 × 3 cell array in collagen (1.0 mg/mL). The field of view is divided into nine zones (I-IX) with dotted lines. Cell protrusions over time are indicated with arrowheads (red: zone I, blue: zone II, green: zone V, white: IX) (Scale bars: 50 μm). (**b**) Immunofluorescence imaging (red: F-actin, blue: Hoechst) of the array if four MCTS that developed from the cells in zone V, VI, VIII, and IX (Scale bar: 50 μm). (**c**) Confocal reflectance imaging shows the collagen I (1.0 mg/mL) microstructure (thickness: 7 μm, maximum projection), and verifies the MCTS were fully embedding when developing (Scale bar: 50 μm). (**d**) 3D rendering of the MCTS array in (**b**) showing single cell migration (arrows) and collective migration (arrowheads) in 3D (Scale bar: 40 μm). The MCTS are color-coded by their volumes.

As discussed previously, the drop-patterned cell arrays enable the study of single-cell behavior in 3D gels over extended periods of time (Fig. 4 and Figure S6). In Fig. 5a, an array of cells was drop-patterned into collagen I and the cells were tracked in nine positions over 3.5 days. Here, we demonstrated the ease of following the progression of multiple cells over periods of time, even when not kept directly on an incubator-equipped microscope stage. Pattern fidelity allowed for easy, repeated location of the cells. The position shift of the collagen gel with respect to the PDMS microwell substrate—the gel detached after cell culture medium was added—did not affect the fidelity of the cell array. For illustration, the region with the 3 × 3 cell array has been visually divided and labeled as zones I through IX. The patterned array consisted of eight single cells (I-VIII) and one doublet (IX). All cells survived, and they divided within two days.

Evidence of protrusive (Fig. 5a, red and blue arrowheads) and motile behaviors was readily observed over the 3.5-day period. Notably, the cells in zones I and II exhibited extensive protrusive behaviors into the collagen matrix starting from day 2, with the protrusions changing directions during the first 7 hours of day 2. Also in the first half of day 2, cells from zones I, III and VII underwent phenotypic change and migrated away from their respective initial positions. After 3.5 days in the collagen gel, cells in zones II, III, IV and VII displayed the highest motility. They continued to proliferate and migrate into neighboring zones.

In contrast, the cells in zones V, VI, VIII and IX were not, at least initially, as motile, however, they did proliferate. The three single cells and one cell doublet began forming MCTS within the first two days. Only after three days did cells from these MCTS begin to form protrusive actions and migrate away from the MCTS in both single-cell (green arrowhead) and collective (white arrowheads) manners.

After 3.5 days, the cells in the 3 × 3 array were fixed and their nuclei (Hoechst, blue) and F-actin (phalloidin, red) were stained. A confocal image stack (height: 112 µm) was acquired from a focal plane above a tumor array to a focal plane below the tumor array (Fig. 5b). Collagen fibers were auto-fluorescent in the blue channel on both the first and the last several images with no F-actin (red) observed, which verified that all the tumors were fully encapsulated in collagen. Reflectance confocal microscopy was used to visualize detailed microstructure of collagen (thickness: 7 μm) surrounding the migrating cells (Fig. 5c). 3D-reconstruction (Fig. 5d) of the array of four solid tumors in Zones V, VI, VIII, and IX clearly shows the spatial relationship between tumors and the cells (marked with arrows) that were escaping the tumors, and reveals that cell migration in collagen did not only happen within the plane of the cell array.

### Longitudinal observations of cell morphology, division, and tumor development in Matrigel

Using collagen I for 3D culturing of cells provides them with a fibrillar network similar to the native ECM in which a mesenchymal phenotype and protrusive sensing are promoted. Matrigel, on the other hand, originating from the epithelial basement membrane, provides a different set of physical and biochemical cues for cells in 3D culture^36,37^, which promote organoid formation and tumor growth^38,39^. As Matrigel is a popular 3D culture matrix, we demonstrated that our drop-patterning method would be amenable to its usage. A Matrigel concentration of 5 mg/mL was used. According to the manufacturer, Matrigel gels rapidly at 22 °C to 37 °C. Indeed, when dropping the cells into Matrigel by inverting the chip at room temperature, we found that before cells reached the coverslip (spacer height: 600 μm), the rapid gelation of Matrigel had already captured the cells in the chamber. This process took only about 10 minutes. After the cells were no longer sinking, the chip was transferred to a 37 °C incubator for 10 minutes to fully gel the matrix. Verification of full cell embedding is shown in Figure S7.

**Figure 6:**
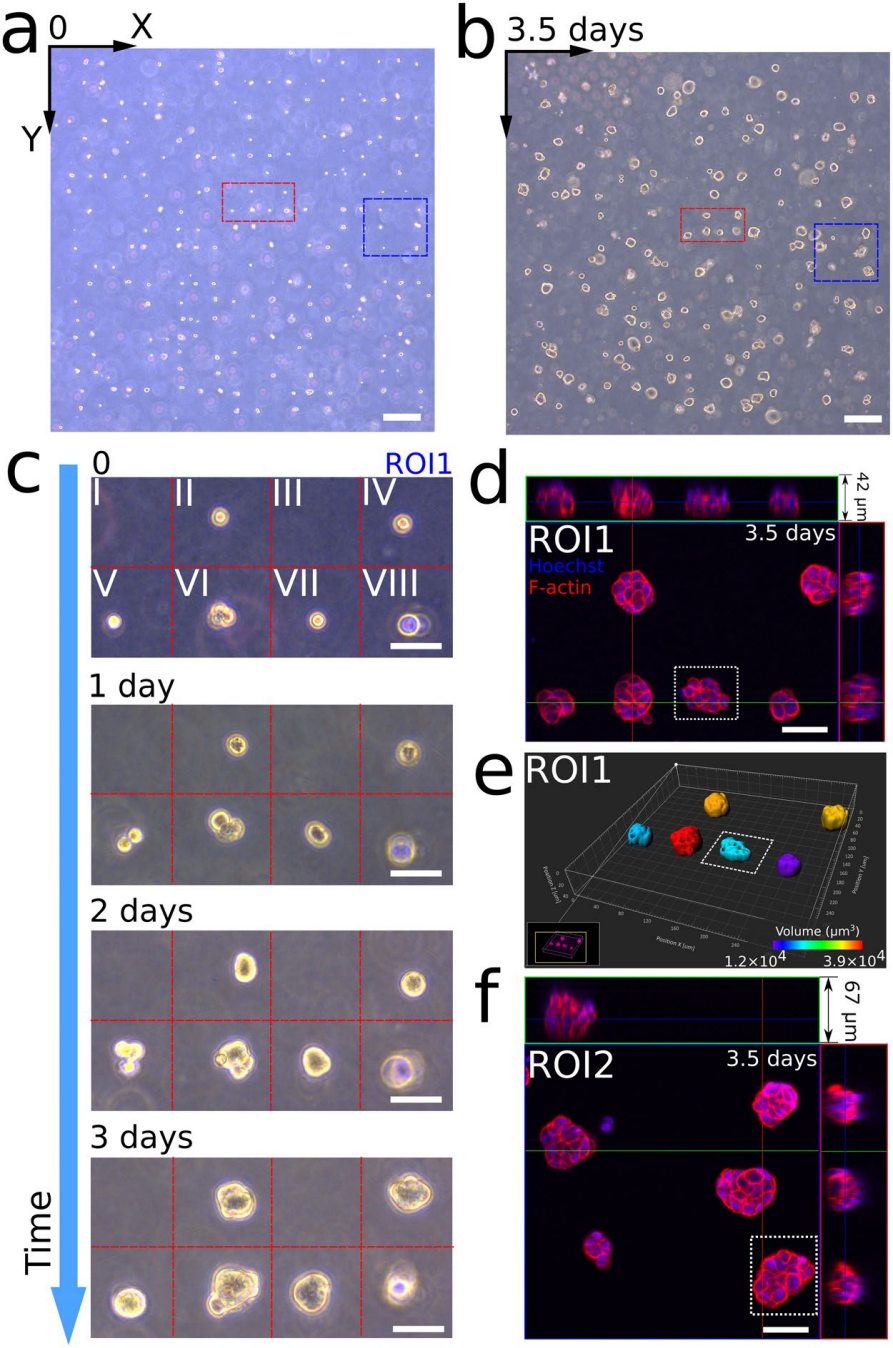
3D on-chip MCTS development in the basement membrane matrix Matrigel. (**a**) Large-scale cell array in Matrigel, which developed into (**b**) an array of MCTS after 3.5 days. Region of interest 1 (ROI1) and region of interest 2 (ROI2) are enclosed by red and blue lines, respectively. (Scale bars: 200 μm) (**c**) Longitudinal observation of tumor development in ROI1 over 3 days. (Scale bars: 50 μm) (**d**) Fluorescence confocal imaging (red: F-actin, blue: Hoechst) of the MCTS array developed from the cells in ROI1 after 3.5 days. (Scale bar: 50 μm) (**e**) 3D rendering of the MCTS array in (**d**) color-coded by volume. (**f**) Fluorescence imaging (red: F-actin, blue: Hoechst) of the MCTS array developed from the cells in ROI2 after 3.5 days. (Scale bar: 50 μm).

Large-scale cell arrays were patterned in Matrigel with a nominal cell-cell spacing of 100 μm (Fig. 6a) and incubated for 3.5 days (Fig. 6b). Within the larger array, two regions of interest are outlined (ROI1: blue and ROI2: red) from which higher magnification insets are presented. The multiple-day monitoring of cell proliferation in ROI1 is shown in Fig. 6c. Again, ROI1 was divided into eight zones. Zone I and III were vacant. The single cells in Zones V and VII divided into doublets after one day. Instead of protruding and migrating through the matrix as in collagen I, the HCT-116 cells tended to maintain their positions and develop into MCTS in the Matrigel. After fixing the tumor arrays in Matrigel at Day 3.5, we acquired fluorescence (red: F-actin, blue: nuclei) confocal images of the solid tumors in ROI1 (Fig. 6d) and ROI2 (Fig. 6f), to further compare the morphologies of these tumors. Figure 6e shows the 3D reconstruction of ROI1, color-coded by tumor volume. From Fig. 6d,f, it is clear that no cells formed protrusions into the Matrigel.

### Other single-cell resolution patterns in 3D

**Figure 7:**
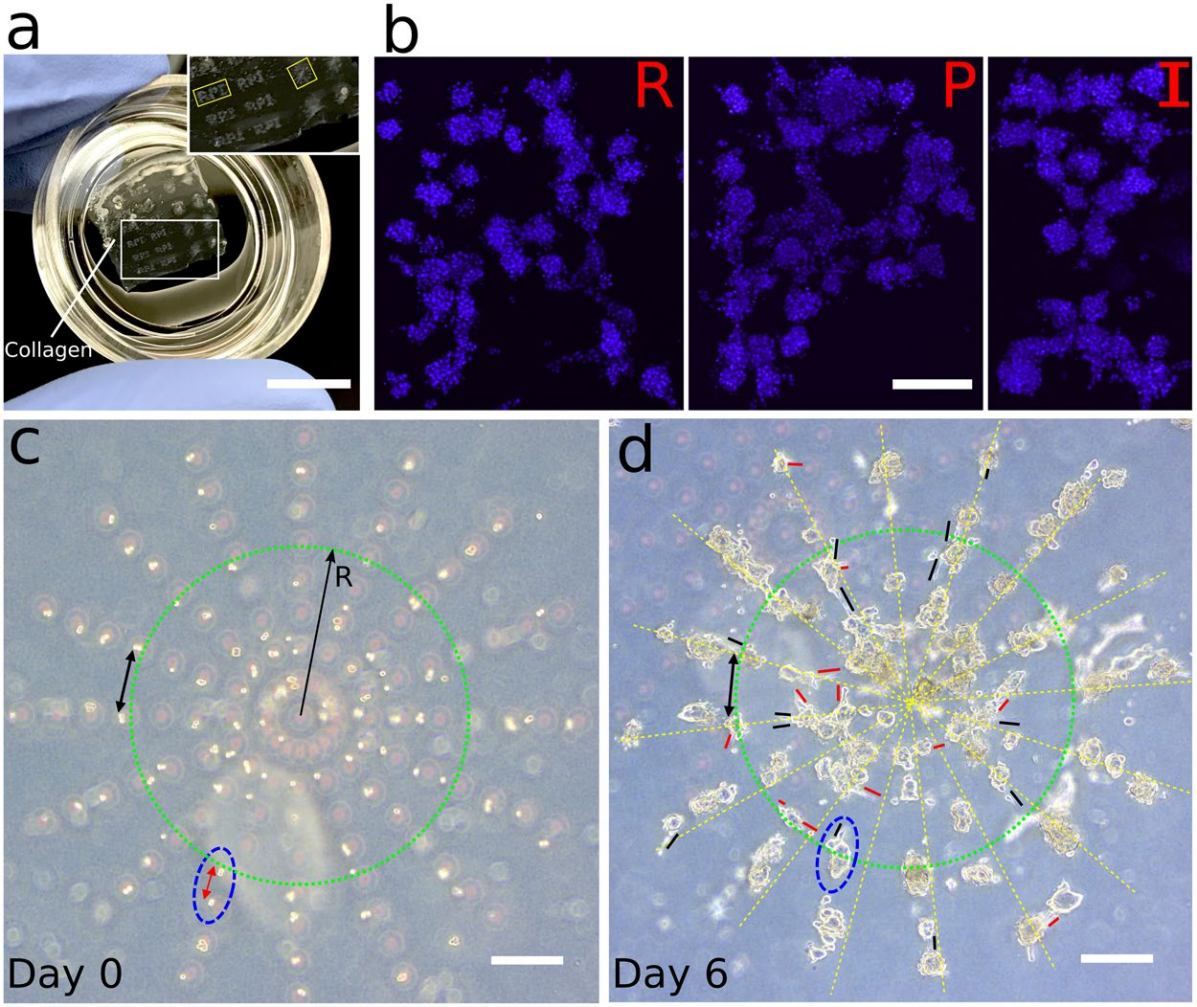
Various patterns at the single-cell level in collagen produced with the drop-patterning method. (**a**) Photograph of the drop-patterned collagen gel in a 35-mm glass-bottomed Petri dish with millimeter-scale tumor patterns growing for six days from the single-cell level. (Scale bar: 15 mm) (**b**) Fluorescence images (Hoechst, maximum projection) of cell-assembled capital letters R, P, and I (abbreviation for Rensselaer Polytechnic Institute) after six days of growth in the collagen. (**c**) Single cells and small cell clusters were drop-patterned in collagen in concentric circles demonstrating the capability of producing varying intercellular spacing within one pattern (double-headed arrows). Ninety-nine of a total of 128 positions were occupied by cells (78% patterning efficiency). (**d**) Six-day culture of the cell pattern in (**c**) showing MCTS growth, cell migration, and cell protrusions in between MCTS (red and black solid lines). The radius R of the green circle is 500 μm. Blue dotted ellipses indicate two separate cells in (**c**) proliferating and merging into one MCTS in (**d**) after six days. (Scale bars: 200 μm).

Array patterns may be an efficient tool for ease of locating individual cells when one studies single cell behaviors in 3D matrices. However, the drop-patterning method may also be used to produce other, more complex patterns that may be designed, for example, to induce or prohibit cell-cell interactions using spatial variations in the cell density. We demonstrated this capability with a couple of different patterns that we dropped into collagen I (Fig. 7a). We illustrate the flexibility in pattern design with the letters R, P, and I (abbreviation for Rensselaer Polytechnic Institute) (Fig. 7b). To demonstrate how variable spacing may be used to investigate cell-cell interaction distances as a function of matrix properties, a concentric-circle pattern consisting of 16 radial lines was drop-patterned and the cells were incubated (Fig. 7c,d) for six days.

In the concentric-circle pattern design, the cell-cell spacing along the radii is 100 μm (red double-headed arrow, Fig. 7c). The cell-cell spacing on a circle is proportional to its radius (i.e., larger when the radius is larger). Thus, the area density of cells varies in radial direction and the cell-cell distance (black double-headed arrows) can be varied within one pattern. We observed that varying cell spacing might be used to probe the sensitivity of cells to signaling from neighboring cells as a function of spacing. Comparing Fig. 7c,d, we found MCTS merged when they were patterned close to each other (blue dotted ellipses). Overlaid on the image in Fig. 7d (at day 6) are the radial patterns (dotted yellow lines) and short solid lines (red and black) parallel to cell protrusions from the tumors. The lengths of the solid lines indicate the protrusion lengths. The black lines represent the cells protruding towards adjacent MCTS in the radial direction, while the red lines indicate protrusions between MCTS in the circumferential direction. The green circle (radius R = 500 μm) with a nominal cell-cell distance (black double-headed arrow) of approximately 200 μm encloses most cell protrusions occurring between MCTS in the circumferential direction (i.e., red solid lines). Cell patterns like the concentric circles may be potentially used as a tool to study cell-cell interactions and characterize the distance range for mechanical or chemical communications between cells with respect to matrix properties.

## Discussion and Outlook

Here we presented the development and performance evaluation of a method for embedding arrays and planar patterns of cells completely within hydrogels. The cells do not sit within discontinuous interfaces or at boundaries between stiffness-mismatched materials. Our desire for developing such a tool arose from facing the challenges of obtaining significant numbers of data points on the physical behavior of single cells and/or aggregates of cells embedded in 3D matrices over time. The drop-patterning method is easy and inexpensive to employ and significantly eases the task of collecting large numbers of data points for many different types of 3D cell studies including: cell-matrix interactions, cell-cell interactions, cell division mechanics, and organoid or MCTS growth.

To evaluate the performance of our method, we drop-patterned cells into collagen I gels using evenly spaced arrays of single-cell-sized microwells. We reported the trapping, patterning, and single-cell efficiencies from hundreds of cells patterned in multiple trials. Reported for similar microwell trapping technologies are trapping efficiencies as high as 85% to 92%^16^. Our experience and other reports, however, indicate that the single-cell trapping efficiency of passive methods such as microwells is highly dependent on cell type and the ease with which the cells may be dissociated. This results in single-cell efficiencies on the order of 26%^40^ to 34%^33^, and probabilities of cell numbers per well or per chamber fitting the Poisson distribution^41^. Our single-cell efficiency is currently on par with these other studies. We believe this may significantly be improved by designing the trapping steps around a more efficient microfluidic method^42,43^.

Matrix materials and their biophysical properties are important considerations when designing and conducting studies of cell behavior in 3D. Depending on the type of study, a model of the mechanical, morphological, and/or biological activity of the ECM may be desired. We demonstrated that the drop-patterning method is applicable to three different types of matrix material: collagen I, Matrigel, and agarose (Figure S9). The results presented here focused on collagen I and Matrigel, but this method may be furthermore expandable to any hydrogel formulation for which the gelation kinetics may be controlled on the time scale of minutes. This provides the basis for which cell-matrix interactions may be studied with respect to many different biochemical compositions, morphologies, and stiffness of ECM at the single-cell level in a more controllable way.

Compared to random mixing of cells in a gel, this well-organized method of 3D cell culture made it possible to keep track of individual cell positions in a large sample size on one chip. Individual MCTS could be easily traced back to the corresponding single cells or cell clusters that they developed from. In a demonstration of the applicability of this method for tracking the manifestation of cell behavioral heterogeneity within a matrix, we point out the distinctly different behaviors of individual cell development in one array in both the collagen I and Matrige. In collagen I, we observed some cells to display more spread and motile behaviors whereas others displayed a more proliferative phenotype (Fig. 5). In Matrigel, although no motile behavior was observed, the morphologies of MCTS (Fig. 6d–f) were distinctly varied. Whereas some MCTS adopted round and smooth boundaries, some MCTS (outlined with dotted lines) were less packed and adopted a “grape-like” morphology. Morphological classifications such as this have been previously described for a panel of human breast cancer cell lines^44^, where the loosely organized grape-like morphology has been attributed to weaker cell-cell adhesion. We believe the drop-patterning chip will become a useful tool to isolate and study the biological basis of different phenotypic behaviors displayed within the same cell line with respect to interactions with matrix materials.

Technologies, such as cell microdroplets^45^ and micro-chambers along a microfluidic channel^33^, are able to provide cells a 3D environment while possibly allowing for cell proliferation to be tracked in individual droplets or chambers. However, these methods lack the ability to expand the complexity of the patterning and accurately control cell-cell interactions. The drop-patterning technique allows for a large degree of flexibility in pattern geometry (Fig. 7) to elicit or minimize various cell interactions via mechanical or chemical cues. We furthermore envision that the drop-patterning method may be used for 3D co-culture studies with, for example, cancer associated fibroblasts, endothelial cells, or immune cells on one chip. Although single-cell microwells and arrays were used here to illustrate the use of this new method, we would like to point out that it is possible to tune the size of the microwells in order to drop-pattern aggregates of cells or pre-produced MCTS.

The flexibility with which one may choose the cell patterns as well as the types of hydrogel makes this a generally useful tool for fields as varied as tissue engineering, stem cell research, *ex vivo* cancer assays, *in vitro* studies on intercellular communication (e.g., neurite outgrowth of primary neurons), and tumor-microenvironment interaction (e.g., tumor angiogenesis). The drop-patterning method may also be used as part of a multiplexed design, integrated with other platforms—such as a chemotaxis device^46^ or an engineered blood vessel^47^—to create a complex engineered niche for large scale studies cell-matrix interactions.

## Materials and Methods

### Design and fabrication of the drop-patterning chip

We designed the microwell patterns on computer aid design (CAD) software SolidWorks (Dassault Systèmes), and all the patterns were printed on a chrome mask by a high-resolution printing service (Front Range Photo Mask, CO, USA). Micro-posts on the silicon wafer were fabricated in a negative photoresist (SU-8 3025, MicroChem, MA, USA) through the techniques of photolithography. In brief, a layer of SU-8 (27.5 μm thick) was spin-coated on a 3-inch silicon wafer for 30 sec at 2,800 rpm. After soft baking at 95 °C for 10 min, the SU-8 coating was crosslinked under the exposure of UV light through a chrome mask. After post-exposure baking at 65 °C for 1 min and 95 °C for 8 min, non-crosslinked SU-8 photoresist was washed off through the developing process, and all the crosslinked micro-posts remained on the silicon wafer. The silicon wafer was then hard baked at 180 °C for 10 min. The heights of these SU-8 features were inspected by a stylus profilometer (Veeco, DekTak 8).

Using soft lithography techniques, we molded microwells onto a PDMS (Sylgard 184, Dow Corning) sheet via the silicon master with micro-posts. First, we treated the surface with Tridecafluoro-1,1,2,2-tetrahydrooctyl-1-trichlorosilane (TFOCS, Gelest, PA, USA) to prevent cured PDMS sticking to the master. Then, we poured a thoroughly mixed, degassed PDMS precursor (ratio of base and curing agent = 10:1 by weight) onto the silicon master in a plastic Petri dish and allowed the PDMS to cure at 70 °C overnight. After the PDMS was fully cured, we peeled it off and cut it into the desired size (25 mm × 25 mm). A PDMS spacer ring (thickness: approximately 600 μm) was directly cut out of a plain PDMS sheet as a square, matching the size of the patterned PDMS substrate. A square coverslip with standard dimensions (22 mm × 22 mm, thickness: 120–160 μm) was used to seal the whole device. Before assembling, we treated the PDMS substrate and spacer ring in a plasma cleaner (Harrick Plasma) and allowed them to partially recover its natural hydrophobicity in a sterile ambient environment overnight. All three components were sterilized all the three components with 70% ethanol and then with UV light for 15 minutes.

### Preparation of collagen gel and Matrigel

Eight parts of type I bovine collagen monomer solution (3.1 mg/mL, pH 2, PureCol, Advanced Matrix, USA) was diluted with one part of 10× PBS, and then neutralized to a pH of 7.2–7.6 with 0.1 M sodium hydroxide (NaOH) solution. To avoid local pH variance, the solution was pipetted up and down each time a fraction of NaOH was added. The final volume was adjusted to ten parts with ultrapure water. Using this method, the concentration of the neutralized collagen solution was 2.48 mg/mL. Based on the final concentration desired, we were able to adjust the collagen solution to any concentration lower than 2.48 mg/mL, by diluting with cell culture medium. Matrigel (growth factor-reduced, Corning Life Sciences, Lowell, MA) was diluted with cell culture medium to a final concentration of 5 mg/mL. To prevent local gelation, all the solutions and tubes were chilled and all mixing operations were conducted on ice, for both gels. Since air could be introduced via mixing process, the final collagen solution was degassed on ice in a vacuum desiccator to eliminate bubbles during gelation.

### Cell culture

In the study of cell proliferation and tumor growth in 3D, we chose a human colon cancer cell line HCT-116 (ATCC) as a cell model. Before loading the cells in the chip, we cultured them on tissue culture flasks in McCoy’s 5 A modified medium (Corning) with 10% (vol/vol) FBS (Gibco) and 1% penicillin/streptomycin (Gibco) at 37 °C and 5% CO_2_ in a humidified incubator. The cell culture medium was changed every other day and passaged when cells reached over 80% confluency. When the cells were embedded in 3D collagen, we continued culturing them by submerging the chip in fresh cell culture medium. Passage numbers of the cells used in this research did not exceed ten.

### Device preparation and assembly

Before drop-patterning, the surface of the inherently hydrophobic PDMS microwell substrate (bottom component, Fig. 1a) was treated with air plasma (Harrick Plasma) for 30 seconds, a process that renders its surface hydrophilic. The substrate was then placed in a sterile ambient environment overnight to partially recover its natural hydrophobicity. This step allowed for optimal wetting behavior while preventing cell attachment to the microwell walls. The surface of the plasma-treated microwell substrate and the glass coverslip were then incubated at room temperature for one hour with a 10% bovine serum albumin (BSA) solution to further prevent cell attachment.

After the BSA treatment, 250 μL cell suspension (1 × 10^6^ cells/mL) was seeded on the microwell substrate and the cells were allowed to settle into the microwells for about 5 min. Then the supernatant was removed and excess, untrapped cells were gently flushed away with phosphate-buffered saline (PBS). A Kimwipe was used to carefully dry the unpatterned area near the edges of the substrate, while keeping the central, patterned area wet. A dry PDMS spacer (middle component, Fig. 1a) was then rapidly placed onto the substrate to create a chamber. This chamber was filled with a collagen solution (1.0 mg/mL) and then sealed with the coverslip (top component, Fig. 1a).

### Immunohistochemistry

HCT-116 tumor arrays were grown in 3D collagen I or Matrigel for multiple days. Collagen gels were then transferred into a glass bottom dish (World Precision Instruments, FL, USA) for fixation and imaging. Because Matrigel tended to break when we tried to transfer it from a chip to a new dish, we directly stained the MCTS in the Matrigel on chip and conducted confocal imaging through the coverslip (Figure S8). Specifically, the samples were washed in PBS, fixed with 3.7% paraformaldehyde at 37 °C for 30 min, and permeabilized with 0.5% Triton X-100 at 37 °C for 30 min. After washing with PBS three times for 30 min, the samples were blocked for 10 hours in 5% BSA in PBS at room temperature. Samples were then incubated at 4 °C with a primary vimentin (Figure S6) antibody (1:50, mouse, Santa Cruz) diluted in incubation solution (PBS with 0.1% BSA) overnight. An F-actin probe rhodamine phalloidin (1:50, R415, Thermo Fisher) and a second antibody (1:50, mouse, Alexa Fluor 488, Santa Cruz) were then applied in dark at 4 °C overnight. The nuclei were then stained with Hoechst (0.2 μg/mL, Hoechst 33342, Thermo Fisher) at room temperature in dark for 4 hours. In the cell viability assays based on collagen, HCT-116 single cells or small cell clusters were stained with calcein AM and EthD-1 (LIVE/DEAD Viability Kit, Invitrogen) 19 hours after drop-patterning.

### Image acquisition and statistical analysis

Bright field images of microwells and cells were obtained with an inverted microscope (Zeiss, Axio Vert.A1). Fluorescence images of cells/MCTS and reflectance imaged of collagen microstructure were acquired with a laser scanning confocal microscope (Zeiss, LSM 510 META). 3D reconstructions of z-stacks of MCTS arrays were performed on software ZEN (Zeiss) and 3D rendering based on confocal microscopic images was made by software Imaris 8 (Bitplane). Cell-cell distances were measured with ImageJ^48^. All data was presented as mean ± standard deviation (s.d.), except for Fig. 3a where the boxes represent the 25th to 75th percentile and the whiskers represent the minimum-maximum range. Statistical difference was determined by Kruskal–Wallis test with Dunn’s post hoc testing for multiple comparisons. Differences were considered significant at *P* < 0.05.

## Electronic supplementary material

Movie S1

Movie S2

Supplementary Information
