# Supplementary material for: Large-scale patterning of single cells and cell clusters in hydrogels
Source: Sci Rep. 2018 Mar 1;8:3849. doi: 10.1038/s41598-018-21989-4 (PMC5832855; doi:10.1038/s41598-018-21989-4)
Supplement: Supplementary file 3 — Supplementary Information [file 41598_2018_21989_MOESM3_ESM.pdf]

Large-scale patterning of single cells and cell clusters in hydrogels

Supplementary Information

Xiangyu Gong[1, 2], and Kristen L. Mills[1, 2,]*

[1]Department of Mechanical, Aerospace, and Nuclear Engineering, Rensselaer Polytechnic Institute, 110 8th St, Troy, NY 12180

[2]Center for Biotechnology and Interdisciplinary Studies, Rensselaer Polytechnic Institute, 110 8th St, Troy, NY 12180

*Correspondence to: millsk2@rpi.edu

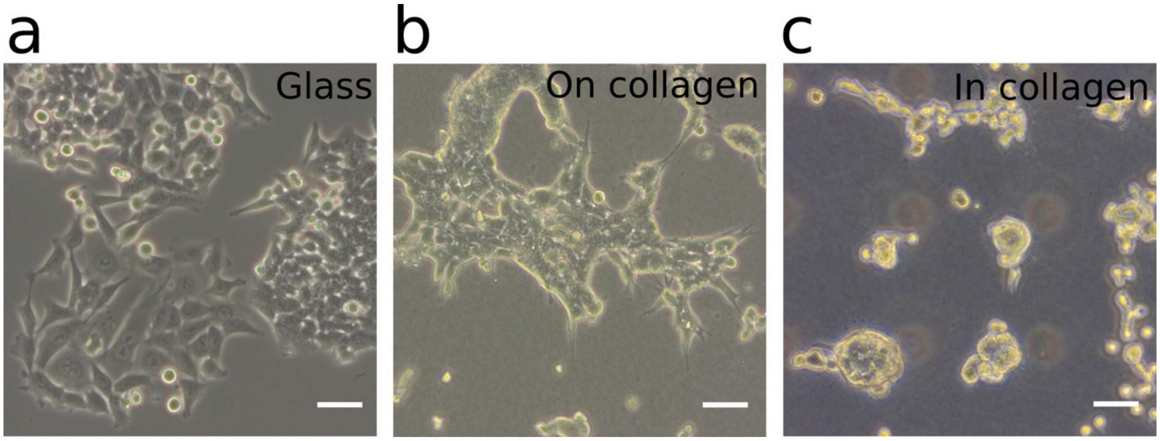

Figure S1. Morphologies of a human colon cancer cell line HCT-116 (a) on a stiff glass surface, (b) on the surface of a collagen gel (1.5 mg/mL), and (c) fully embedded in 3D collagen gel (1.0 mg/mL). Cells proliferate and form a monolayer on 2D substrates. When fully embedded in collagen gel, cells form aggregates or multicellular tumor spheroids. Scale bars are 50 μm.

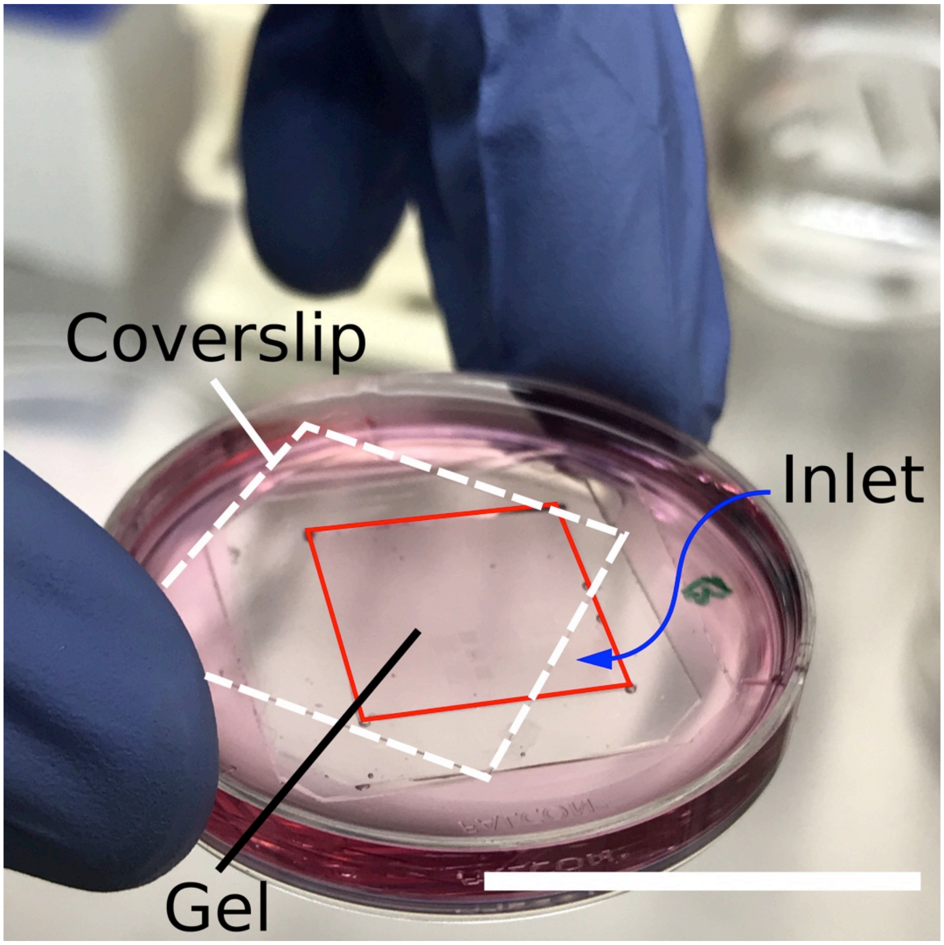

Figure S2. Close view of a drop-patterning chip that was submerged in cell culture medium in a petri dish lid. A gel with cell patterns was sealed in the chip. The glass coverslip was slid aside to create an inlet for nutrient and oxygen. Scale bar: 25 mm.

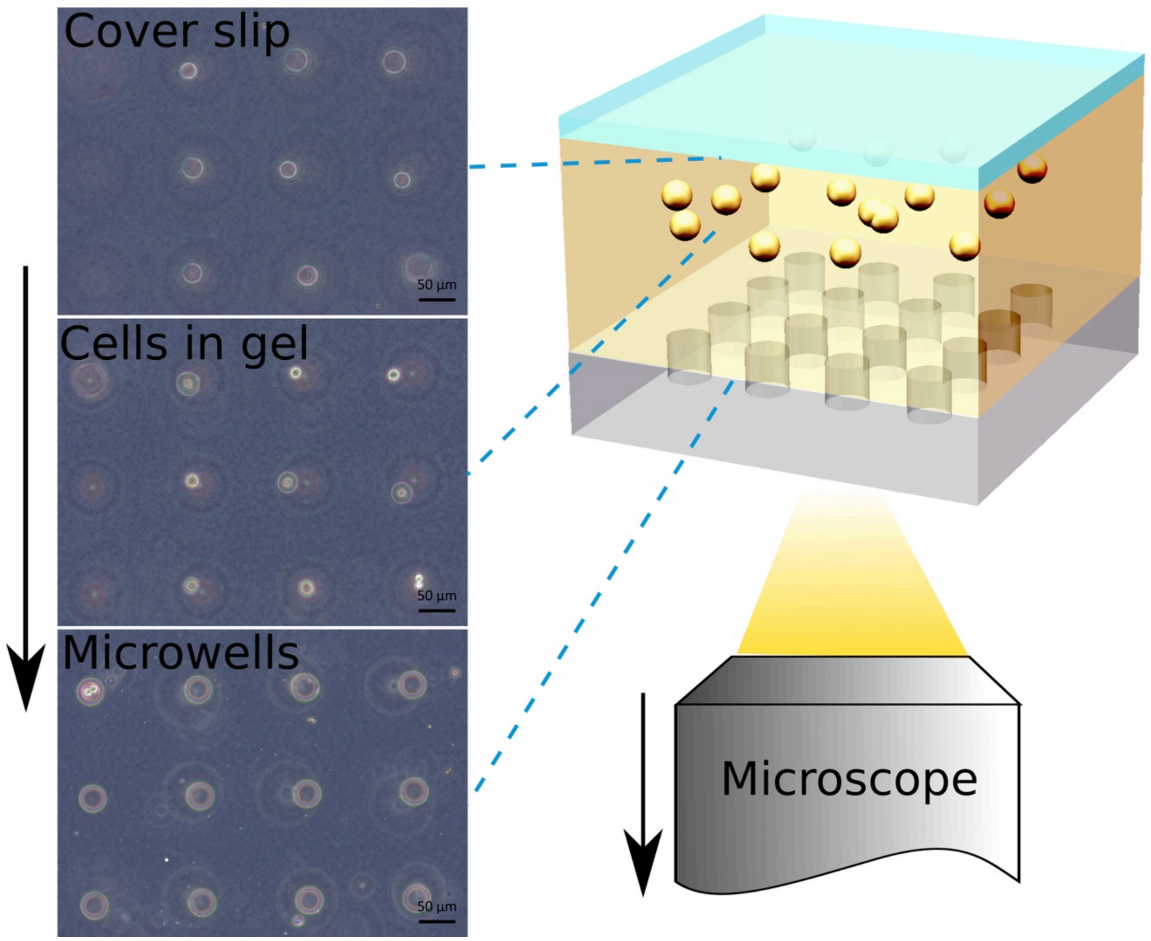

Figure S3. Verification of cell spatial distribution in collagen gel. Using a light microscope (Zeiss, Axio Vert.A1), we verified that the cell array had been fully embedded in collagen gel (Movie S2). While the focal plane was moving down, multiple images of a fixed field of view were acquired. From the coverslip to microwell substrate, the multiple-cell array was out of focus at the beginning, then became focused, and out of focus again, which suggests that the cells were embedded in the gel between the coverslip and microwell substrate. This simple method was used to verify all the drop-patterned cells in this work.

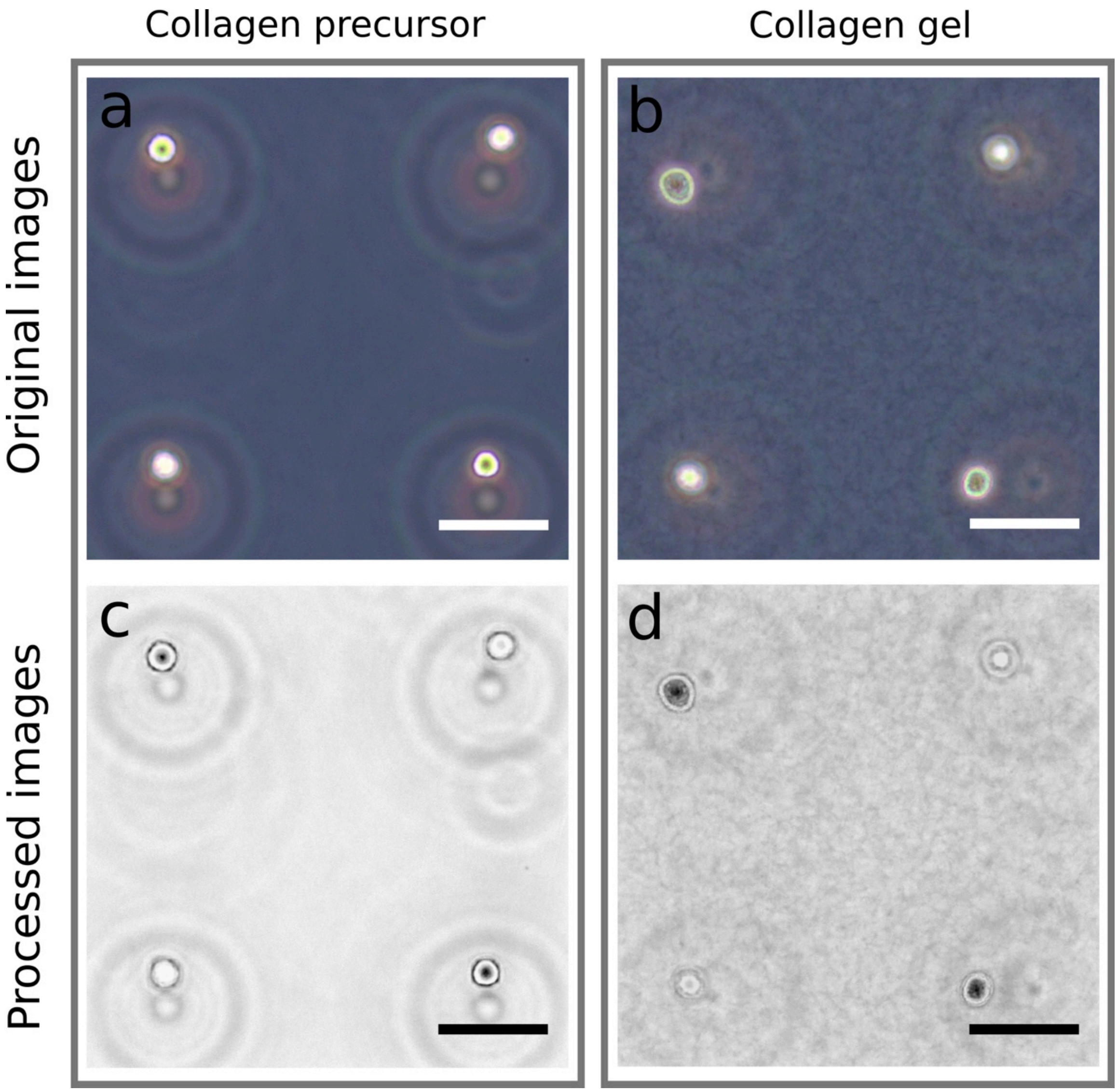

Figure S4. The states of collagen (1.0 mg/mL) before (a, c) and after (b, d) gelation. (a, b) We imaged the same region of a drop-patterning chip before (a: precursor) and after (b: gel) inversion and gelation in a 37 °C incubator. (c, d) ImageJ subtracted the background and enhanced the contrast of the original images, to better visualize the structural difference between collagen precursor and collagen gel. Compared to the precursor (c), collagen fibrillar structure (d) was observed to be surrounding the embedded cells. Scale bars are 50 µm.

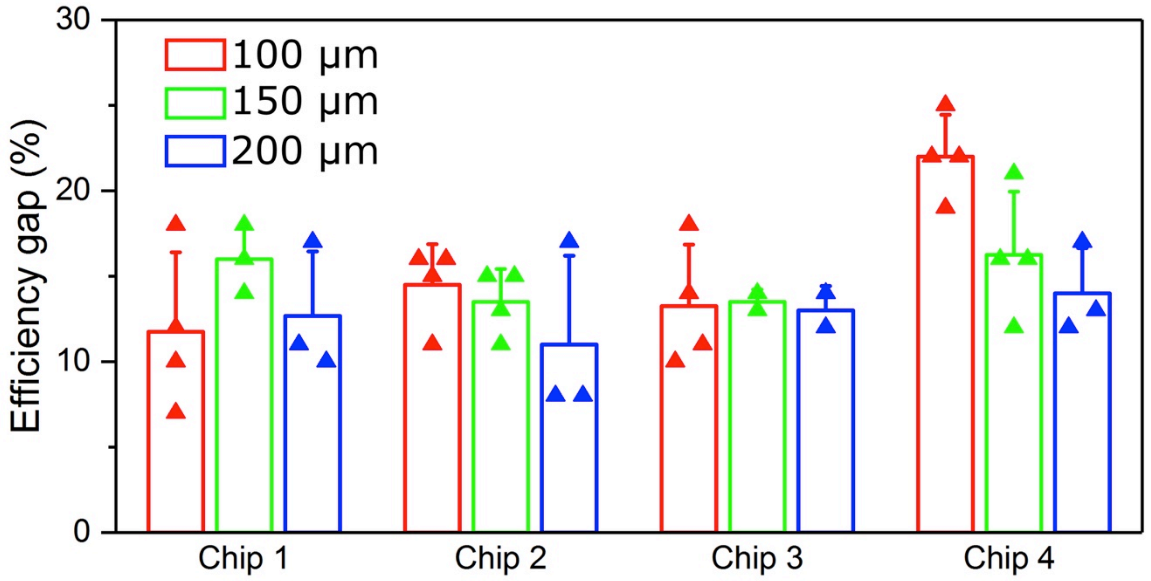

Figure S5. Statistics of the discrepancy between trapping efficiency and patterning efficiency of each array on each chip. Each data point represents a trapping efficiency subtracted by the corresponding patterning efficiency of an array. This discrepancy suggests 10-25% occupancy, on average, of the 400 positions in an array could be lost because a fraction of the trapped cells were stuck in the microwells. Kruskal-Wallis test with Dunn's post hoc testing was conducted to compare different chips with the same cell-cell distances, and different inter-well distances on the same chips. There was no statistical difference at $P < 0.05$.

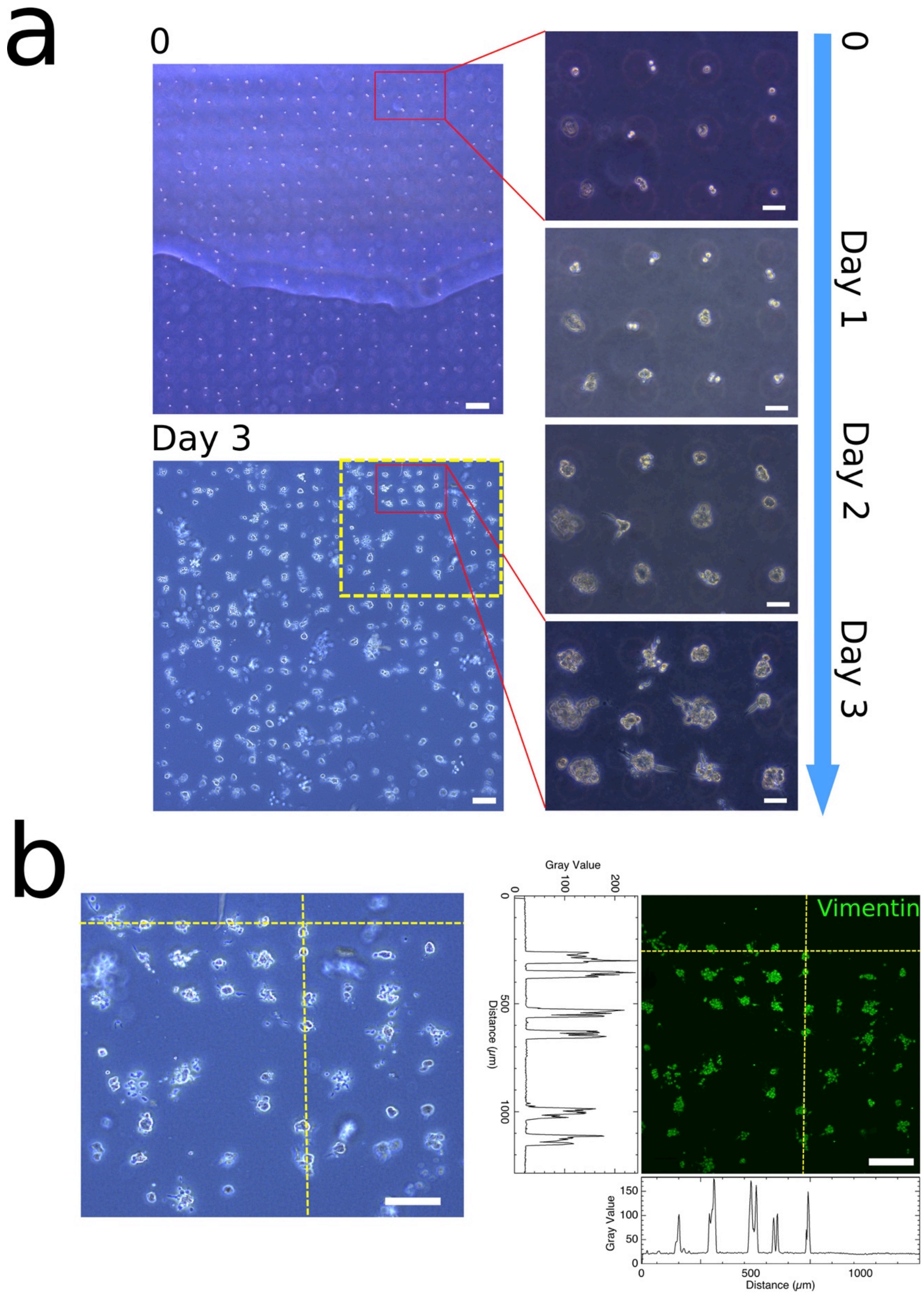
a
0
Day 3
0
Day 1
Day 2
Day 3
b
Gray Value
0
100
200
Distance (μm)
500
1000
Vimentin
150
100
50
0
Gray Value
0
500
1000
Distance (μm)

Figure S6. (a) Large-scale cell array patterning in 3D collagen (Left, scale bars: 200 μm) with ROI1 (highlighted in red, corresponding to Figure 4) for cell behavior tracking over three days (Right, scale bars: 50 μm). (b) Bright-field imaging of the region indicated by yellow dotted lines in (a), and immunofluorescence confocal imaging (maximum projection) of vimentin (staining protocol in Materials and Methods) in the cells of the same region. Fluorescence intensity (grey value) was measured along the horizontal and the vertical lines indicated in the images (scale bars: 100 μm). We demonstrated the robust staining for the samples prepared on the drop-patterning chip and the ease of analyzing the expression in individual positions.

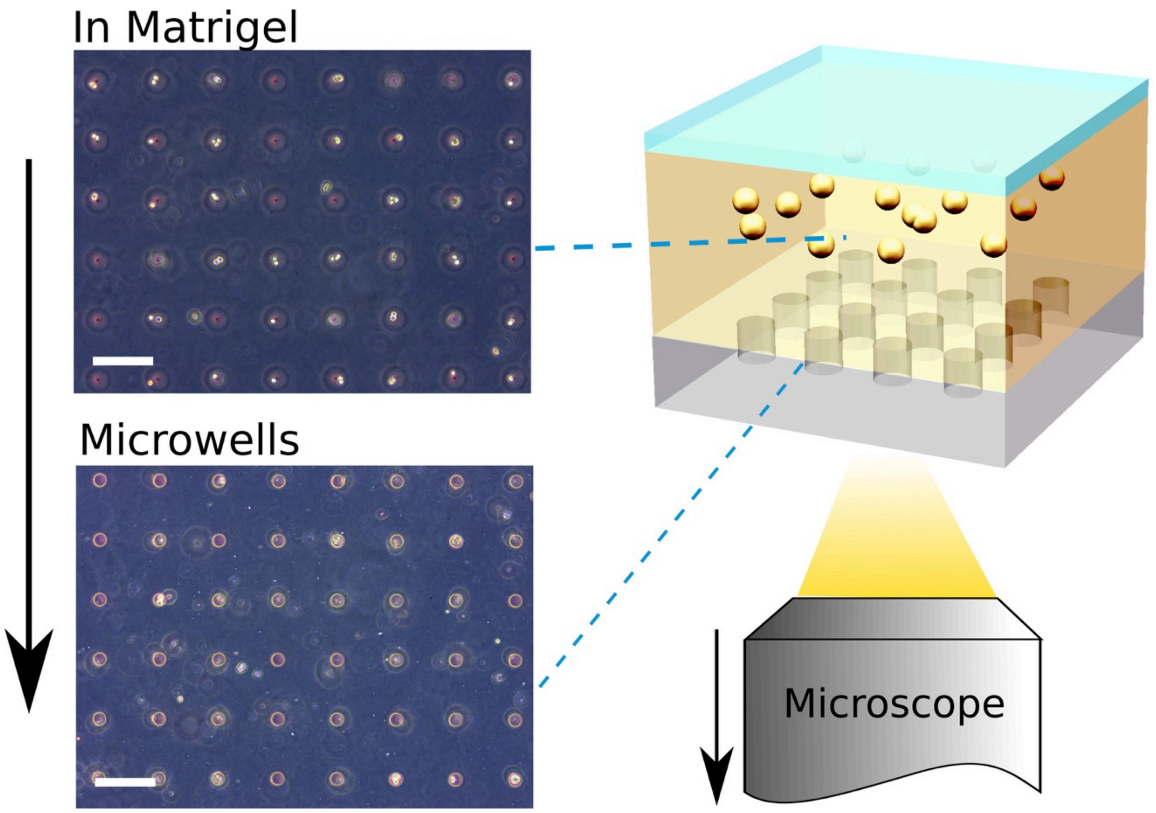

Figure S7. Verifying cells were fully embedded in Matrigel. While the focal plane was moving down, the cell array became focused, and then out of focus when the microwell substrate became focused, which suggests that the cells were embedded in the gel between the coverslip and microwell substrate. (Scale bars: 150 μm)

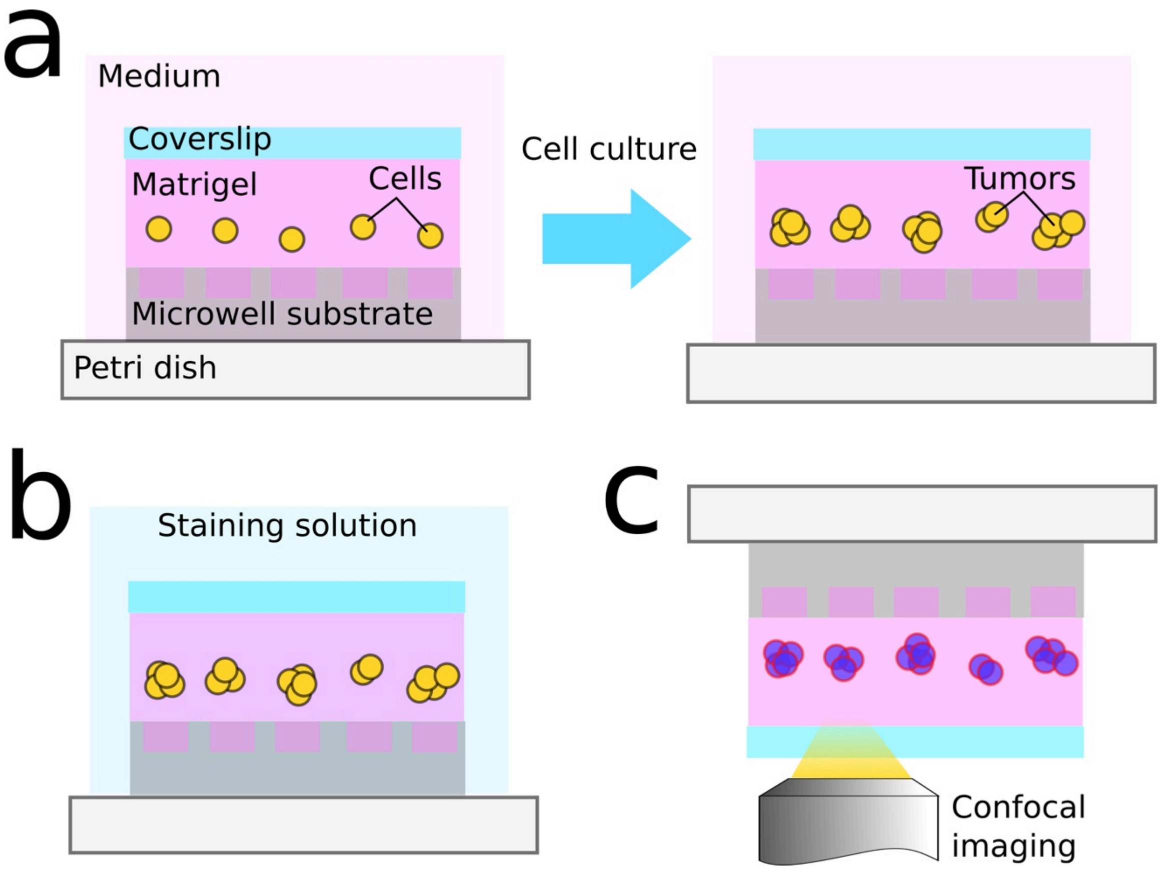

Figure S8. On-chip immunofluorescence staining and confocal imaging. (a) Cell arrays in Matrigel at the single-cell level grew into tumors after multiple-day culture. (b) Fixing and staining solutions were allowed to diffuse into Matrigel through the nutrient inlet. (c) Solution was removed after finishing cell staining. The chip was inverted and confocal images were taken through the coverslip on the chip.

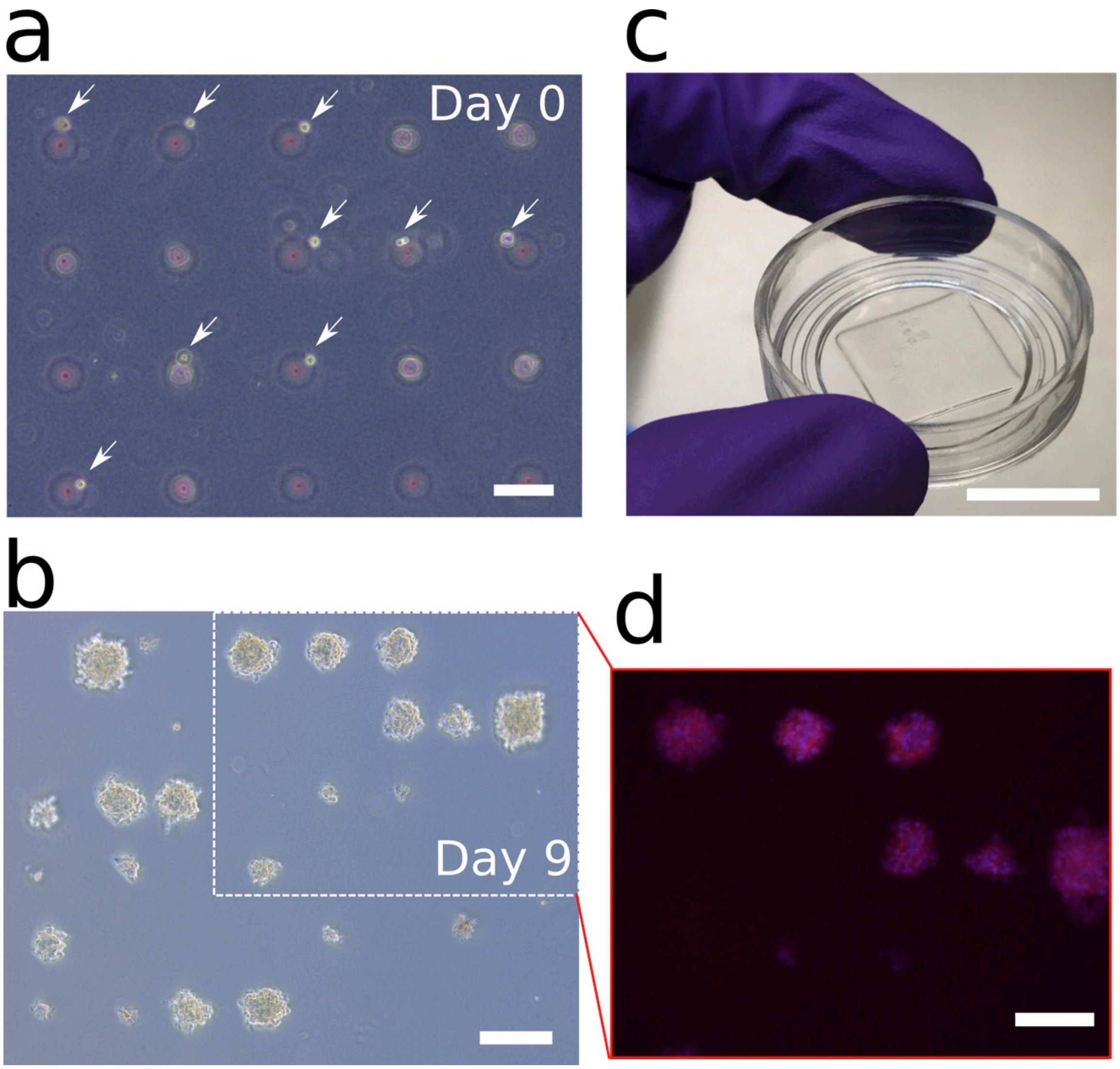

Figure S9. Drop patterning in agarose gel. (a) Cell patterning of HCT-116 (indicated with arrows, scale bar: 100 μm) in 3D agarose (0.3% w/v) at the single cell level developed into (b) an array of tumor after 9 days (scale bar: 200 μm). The enclosed region in (b) is the same region presented in (a). (c) Photograph of the agarose gel transferred in a 35-mm glass bottom petri dish with millimeter-scale tumor patterns growing for 9 days from the single-cell level (Scale bar: 15 mm). (d) Immunofluorescence imaging (blue: Hoechst, red: F-actin, maximum projection) of the enclosed region in (b) (Scale bar: 200 μm).

Supplementary Movies

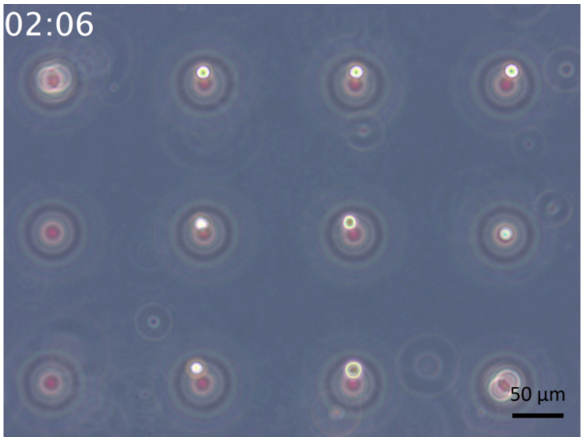

Movie S1. A cell array falling through collagen precursor from microwells to the glass coverslip on a drop-patterning chip.

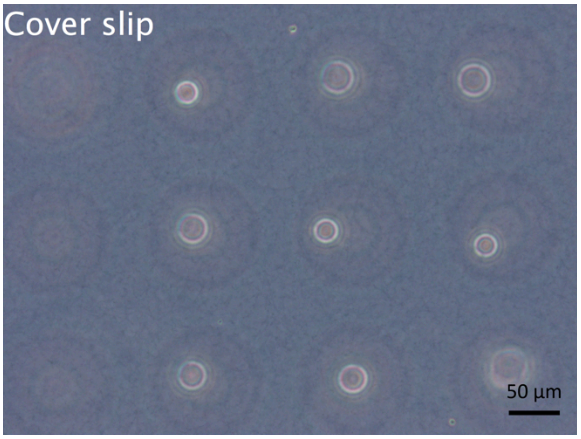

Movie S2. Verification of fully embedding of cells via drop-patterning method.
